# Urinary Biomarkers TIMP-2 and IGFBP7 Early Predict Acute Kidney Injury after Major Surgery

**DOI:** 10.1371/journal.pone.0120863

**Published:** 2015-03-23

**Authors:** Ivan Gocze, Matthias Koch, Philipp Renner, Florian Zeman, Bernhard M. Graf, Marc H. Dahlke, Michael Nerlich, Hans J. Schlitt, John A. Kellum, Thomas Bein

**Affiliations:** 1 Department of Surgery, University Medical Center Regensburg, Franz-Josef-Strauss-Allee 11, 93053 Regensburg, Germany; 2 Department of Trauma Surgery, University Medical Center Regensburg, Franz-Josef-Strauss-Allee 11, 93053 Regensburg, Germany; 3 Center for Clinical Studies, University Medical Center Regensburg, Franz-Josef-Strauss-Allee 11, 93053 Regensburg, Germany; 4 Department of Anesthesiology, University Medical Center Regensburg, Franz-Josef- Strauss-Allee 11, 93053 Regensburg, Germany; 5 Center for Critical Care Nephrology and CRISMA (Clinical Research, Investigation, and Systems Modeling of Acute Illness) Center, Department of Critical Care Medicine, University of Pittsburgh, Pittsburgh, PA 15621, United States of America; University Hospital Münster, GERMANY

## Abstract

**Objective:**

To assess the ability of the urinary biomarkers IGFBP7 (insulin-like growth factor-binding protein 7) and TIMP-2 (tissue inhibitor of metalloproteinase 2) to early predict acute kidney injury (AKI) in high-risk surgical patients.

**Introduction:**

Postoperative AKI is associated with an increase in short and long-term mortality. Using IGFBP7 and TIMP-2 for early detection of cellular kidney injury, thus allowing the early initiation of renal protection measures, may represent a new concept of evaluating renal function.

**Methods:**

In this prospective study, urinary [TIMP-2]×[IGFBP7] was measured in surgical patients at high risk for AKI. A predefined cut-off value of [TIMP-2]×[IGFBP7] >0.3 was used for assessing diagnostic accuracy. Perioperative characteristics were evaluated, and ROC analyses as well as logistic regression models of risk assessment were calculated with and without a [TIMP-2]×[IGFBP7] test.

**Results:**

107 patients were included in the study, of whom 45 (42%) developed AKI. The highest median values of biomarker were detected in septic, transplant and patients after hepatic surgery (1.24 vs 0.45 vs 0.47 ng/l^2^/1000). The area under receiving operating characteristic curve (AUC) for the risk of any AKI was 0.85, for early use of RRT 0.83 and for 28-day mortality 0.77. In a multivariable model with established perioperative risk factors, the [TIMP-2]×[IGFBP7] test was the strongest predictor of AKI and significantly improved the risk assessment (p<0.001).

**Conclusions:**

Urinary [TIMP-2]×[IGFBP7] test sufficiently detect patients with risk of AKI after major non-cardiac surgery. Due to its rapid responsiveness it extends the time frame for intervention to prevent development of AKI.

## Introduction

Postoperative acute kidney injury (AKI) is one of the most common postoperative complications and is associated with an increase in hospital mortality and decreased survival for up to 15 years after surgery [1–5]. The prognosis of patients with AKI is still poor, intervention for prevention and therapy of AKI are currently only initiated in the late phase of already established injury; therefore, benefits remain limited [6].

Using biomarkers for the early detection of cellular injury, thus allowing the early initiation of renal protection measures, may represent a new concept of evaluating renal function in critically ill patients [7,8]. Combination of two novel urinary cell-cycle arrest biomarkers, i.e. the insulin-like growth factor-binding protein 7 and the tissue inhibitor of metalloproteinase-2 ([TIMP-2]×[IGFBP7] panel) was described and validated in two multicenter studies for prediction of risk of moderate and severe AKI (AKI stage 2 and 3 according to KDIGO 2012 classification) in critically ill patients. Both TIMP2 and IGFBP7 are markers of cellular stress in the early phase of tubular cell injury caused by a wide variety of insults (inflammation, ischemia, oxidative stress, drugs, and toxins) [9–12]. Therefore, both markers are involved in the process of G1 cell-cycle arrest that prevents cells from dividing in the case of damage to the DNA until such damage can be repaired [13]. Importantly, both biomarkers appear as “alarm” proteins for other nearby cells in a paracrine fashion [14,15]. These two biomarkers performed better in prediction of AKI than NGAL (AUC 0.64), KIM-1 (AUC 0.69), IL-18 (AUC 0.76), L-FABP (AUC 0.66), or Cystatin C (AUC 0.63) [16,17]. In cardiac surgery patients act [TIMP-2]×[IGFBP7] as a sensitive predictor of AKI and may help to predict renal recovery after AKI using cutoff of 0.5 [18].

The aim of the current study was to evaluate, how the biomarker would perform in non-cardiac surgical patients, if assessed in very early phase after surgery by using a validated cutoff of > 0.3. Moreover, we investigated the performance of the [TIMP-2]×[IGFBP7] test across different severities of AKI (including or excluding stage 1). In addition to perioperative risk factors, we assessed the risk stratification in conjunction with bedside clinical parameters at the time of biomarker assessment. Finally we tested the relation between early cell cycle arrest in the kidney and outcome in surgical patients.

## Methods

### Patients

**Fig 1:**
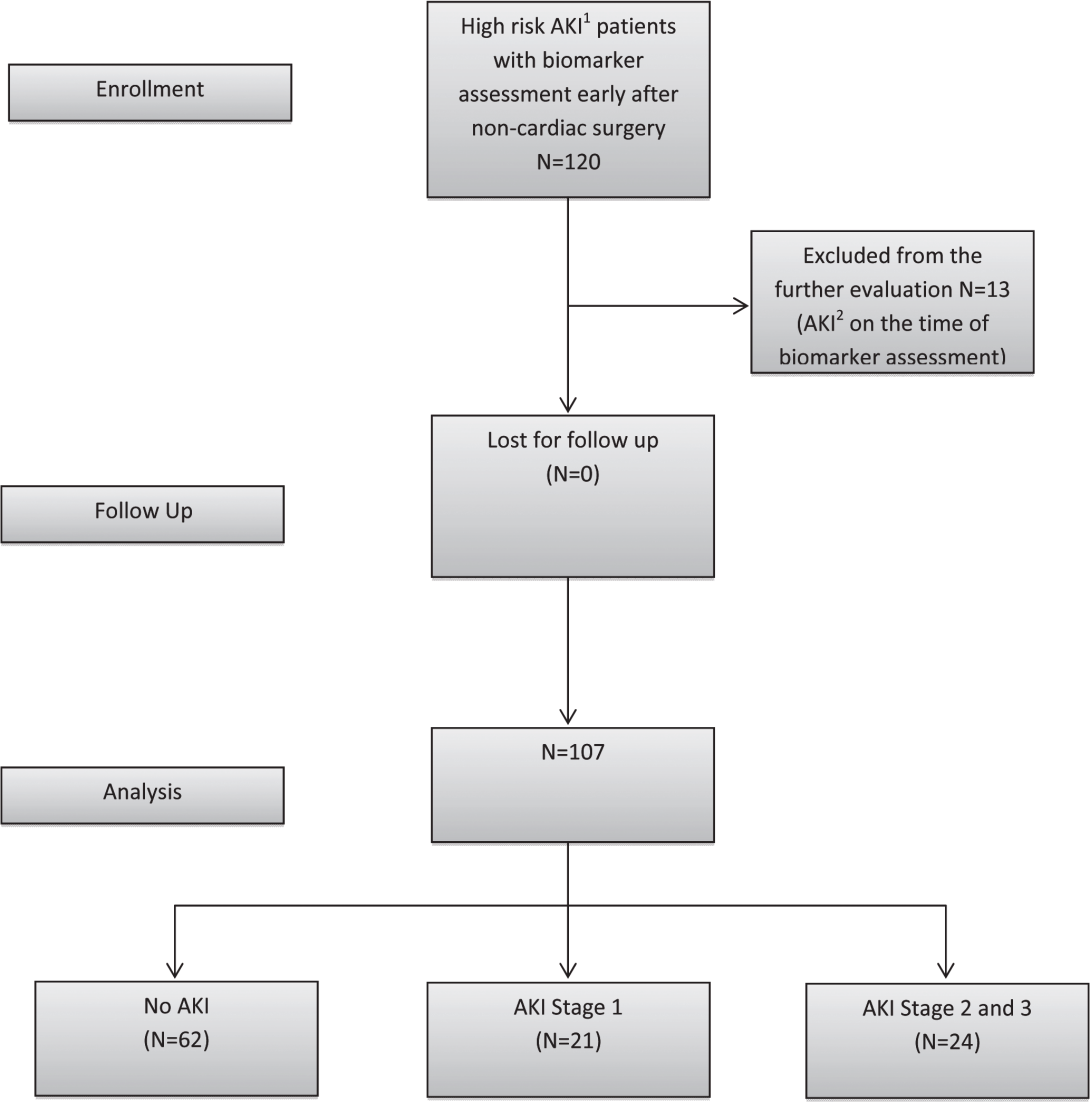
Study design and flow diagram. ^1^ High Risk for AKI—major surgery and one additional risk factor—critical illness, sepsis, major trauma, chronic renal disease or use of radiocontrast agent.^2^ AKI was defined according to KDIGO 2012 criteria by creatinine increase of > 0.3 after surgery.

120 patients were evaluated between May 2013 and November 2013 in the multidisciplinary surgical ICU of a tertiary care university hospital; 13 patients were excluded because they had already developed AKI before biomarker assessment (based on an increase in SCr >0.3 after surgery) (Fig. 1). The study was approved by the local Institutional Review Board (Ethics Committee, University of Regensburg, no. 13-101-0191). Written consent was obtained from all patients or from their next of kin. All patients over the age of 18 years, who had received major non-cardiac surgery, were transported to the ICU immediately after the completion of surgery. Additionally, the patients had at least one risk factor for AKI, such as trauma, sepsis, critical illness, chronic renal disease, and use of an intravenous radiocontrast agent and were thus eligible for inclusion into the study. Critical illness was defined as requirement of inotropic support or mechanical ventilation at the time of admission to the ICU. Patients were excluded if they had end-stage renal disease or developed AKI during the period of time until biomarker assessment.

### Measurements

Urine samples for biomarker assessment were taken from the urinary catheter of eligible patients soon after the transfer from the operating theatre to the ICU. The level of [TIMP-2]×[IGFBP7] was measured by means of the immunoassay method integrated in the Astute 140 Meter Kit (Astute Medical Inc., San Diego, CA, USA). All values for [TIMP-2]×[IGFBP7] are reported in units of (ng/mL)^2^ /1000. In accordance with the validation study, the cut-off of [TIMP-2]×[IGFBP7] >0.3 was used for predicting AKI [16]. The incidence and severity of AKI based on SCr, UO, and provision of RRT were assessed over the first 48 h after admission. SCr was measured before surgery, at admission to the ICU, and then daily during the ICU stay. UO was assessed hourly during the first 48 h. In addition, we recorded different variables, such as type of surgery or diagnosis at admission, the Simplified Acute Physiology Score II (SAPS-II) at admission, age, sex, weight (kg), height (m), creatinine level and norepinephrine dosage (μg/kg/min) at ICU admission, mean arterial pressure (MAP), hemoglobin level (g/dL), cumulative fluid balance (mL/24 h), and urine production (mL/kg/h) at the time of biomarker assessment.

### Statistical methods

Continuous variables are presented as a mean (standard deviation [SD]), and categorical data as frequency counts (percentages). [TIMP-2]×[IGFBP7] values are presented as median values (interquartile ranges [IQR]) and as range because of the skewed distribution of the data. Continuous variables were compared by the Student's *t* test or the Mann-Whitney *U* test, depending on the distribution of the observed data. Receiver-operating characteristic (ROC) analyses were conducted to differentiate between patient groups ((all stages of AKI [yes/no], moderate or severe AKI [yes/no], RRT [yes/no], and ICU mortality [yes/no]), and the optimal cutoff was estimated according to the Youden Index. Estimates for the area under the curve (AUC) with the corresponding 95% confidence interval (CI) were reported as well as sensitivity and specificity. Multivariable logistic regression models were calculated by means of odds ratios (OR’s) and corresponding 95% CI’s to assess the predictive ability of [TIMP-2]×[IGFBP7] for AKI development, for use of RRT and for 28-day mortality. To compare the fit of two nested models, we calculated AUCs of the predicted probabilities and conducted a likelihood-ratio test. A value of *P* ≤0.05 was considered to indicate statistical significance. All analyses were done with IBM SPSS Statistics 21.0.0.1 and R (version 3.0.2).

## Results

### Baseline and clinical characteristics; ROC analysis

**Fig 2:**
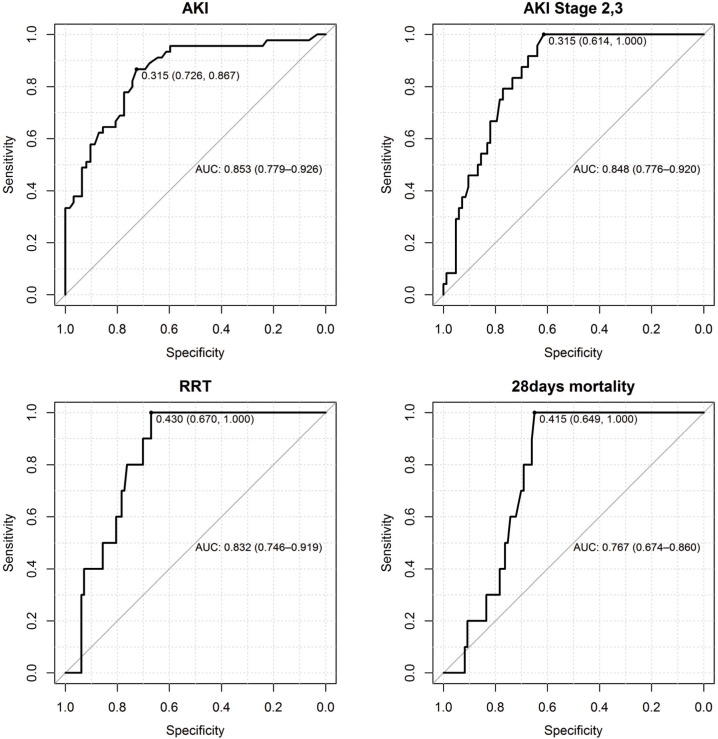
Area under the curve (AUC) and the best cut-offs for predicting AKI, AKI Stage 2 and 3, early use of RRT and 28-days mortality.

The baseline characteristics of the patients are shown in Table 1. The mean time between admission to the ICU and biomarker assessment was 245 minutes (SD 152). 45 (42%) patients developed AKI in the first 48 h of their ICU stay, 24 (22%) patients had moderate and severe AKI (stage 2 and 3), and 10 (9%) patients required RRT within the first 48 h after admission. 8 (8%) patients died in the ICU and 10 (9.3%) patients within the first 28 days after surgery. In the hepatobiliary subgroups of patients 1 of 12 developed AKI, 6 of 14 transplant patients, 4 of 13 cancer patients, 16 of 33 after vascular surgery, 9 of 21 major trauma patients and 8 of 10 septic patients (Table 2). The median value of [TIMP-2]×[IGFBP7] in patients without AKI was 0.19 (IQR 0.1, 0.34); in patients with AKI stage 1: 0.51 (IQR 0.38, 2.66); 1.24 (IQR 0.56, 3.00) in patients with stage 2 and 3; and 1.35 (IQR 0.76, 3.39) in patients who subsequently received RRT. The RRT was started in the meantime of 22.06 hours (SD 14.8) after biomarker assessment and < 48 hours after ICU admission (in 5 patients due to refractory hyperkalemia, in 1 patient due to severe acidosis and in 4 patients due to oliguria < 0.3 ml/kg/h for > 24hours). The AUC for predicting AKI (all stages) was 0.85 (95% CI 0.78, 0.93) and 0.85 (95% CI 0.78, 0.92) for predicting AKI stage 2 and 3, 0.83 for the early use of RRT and 0.77 for 28-day mortality (95% CI 0.67, 0.80) (Fig. 2).

**Table 1:** Patient characteristics (n = 107).

**Baseline characteristics**	**Mean (SD)**	
Age (years)	60.03 (14.78)	
BMI (kg/m^2^)	27.45 (5.64)	
Weight (kg)	81.49 (18.26)	
SAPS II	22.13 (9.63)	
ICU (days)	9.50 (14.05)	
Chronic kidney disease	18 (15%)	
Creatinine at admission ICU	1.13 (0.60)	
AKI	45 (42%)	
AKI stage 2 and 3	24 (22%)	
RRT <48 hours after admission	10 (9%)	
28-day mortality	10 (9%)	
**Diagnosis/Surgery at admission**	**Frequency (percentage)**	(IGFBP-7)*(TIMP-2) values (Median (IQR), min-max)
Hepatobiliary	12 (11%)	0.46 (0.30, 0.78), 0.22–1.78
Transplant	14 (13%)	0.45 (0.15, 1.50), 0.04–4.18
Cancer	13 (12%)	0.22 (0.11, 1.99), 0.02–9.50
Vascular	33 (31%)	0.27 (0.13, 0.36), 0.02–0.87
Severe Trauma	21 (20%)	0.38 (0.11, 1.27), 0.04–9.59
Sepsis	10 (9%)	1.24 (0.29, 2.83), 0.08–5.91
Other	4 (4%)	
(IGFBP-7)*(TIMP-2) values	**Median (IQR), min-max**	
Ø AKI	0.19 (0.10, 0.34), 0.02–1.79	
AKI Stage 1	0.51 (0.29,2.29), 0.04–9.50	
AKI Stage 2 and 3	1.24 (0.56, 3.00), 0.32–9.59	
RRT <48 hours after admission	1.35 (0.76, 3.39), 0.44–4.18	

BMI, body mass index; SAPS II, Simplified Acute Physiology Score II; ICU, intensive care unit; AKI, acute kidney injury; RRT, renal replacement therapy; SD, standard deviation; IGFBP7, insulin-like growth factor-binding protein 7; TIMP-2, tissue inhibitor of metalloproteinase.

**Table 2:** Baseline characteristics of the study subgroups.

	**Hepatic (n = 12)**	**Transplant (n = 14)**	**Cancer (n = 13)**	**Vascular (n = 33)**	**Trauma (n = 21)**	**Sepsis (n = 10)**
**Age**	61.75 (SD 15.33)	54.50 (SD 11.31)	59.23 (SD 12.58)	64.55 (SD 9.42)	54.71 (SD 23.13)	64.30 (SD 9.66)
**AKI yes**	1 (8%)	6 (43%)	4 (31%)	16 (49%)	9 (43%)	8 (80%)
**[TIMP2xIGFBP7]>0.3**	9 (75%)	8 (57%)	5 (39%)	14 (42%)	13 (62%)	8 (80%)
**SAPS II**	15.58 (SD 8.68)	20.14 (SD 9.17)	18.90 (SD 4.83)	23.94 (SD 9.52)	23.71 (SD 8.14)	31.80 (SD 9.67)
**Fluid balance (ml)**	1419 (SD 1170)	1121 (SD 902)	939 (SD 1122)	1845 (SD 1265)	1518 (SD 1572)	2942 (SD 2698)
**MAP (mmHg)**	77.61 (SD 12.36)	81.32 (SD 11.82)	77.61 (SD 10.74)	78.61 (SD 8.09)	77.11 (SD 11.71)	76.99 (SD 5.09)
**Hemoglobin (g/dl)**	10.97 (SD 1.82)	9.57 (SD 1.52)	10.01 (SD 2.17)	9.80 (SD 1.60)	9.01 (SD 1.17)	9.14 (SD 1.38)
**Urine Output (ml/kg/h)**	1.28 (SD 0.64)	1.29 (SD 0.89)	1.47 (SD 0.72)	1.31 (SD 0.62)	1.68 (SD 0.84)	1.05 (SD 0.73)

Data are mean (SD) or n (%). Fluid 24 = Fluid balance in the first 24 hours after ICU admission; MAP = mean "mean arterial pressure" over the first 24 hours; Hemoglobin = mean hemoglobin level in the first 24 hours; Urine output = mean urine output ml/kg/h in first 24 hours.

### Bedside perioperative characteristics and postoperative clinical variables with and without the [TIMP-2]×[IGFBP7] test

Table 3 shows the performance of the [TIMP-2]×[IGFBP7] test in combination with established perioperative risk factors for AKI, such as age, severity of illness score SAPS II, and creatinine level at ICU admission. Addition of biomarkers significantly improved the risk assessment of AKI; AUC increased from 0.72 (95% CI 0.62, 0.81) to 0.88 (0.82, 0.94), p<0.001, and AKI Stage 2 and 3 AUC 0.81 (0.70, 0.90) improved to 0.87 (0.79, 0.95), p<0.001.

**Table 3:** Multivariable logistic regression model of perioperative parameters at the time of biomarker assessment with and without the (TIMP-2)*(IGFBP7) for risk assessment of AKI, AKI Stage 2 and 3, RRT and 28-day mortality.

	AKI		RRT		28 days mortality		AKI 2, 3	
Variable	OR (95%-CI)	p-value	OR (95%-CI)	p-value	OR (95%-CI)	p-value	OR (95%-CI)	p-value
Age (per year)	1.02 (0.98, 1.05)	.400	1.03 (0.97, 1.11)	0.332	1.01 (0.96, 1.06)	0.757	1.00 (0.96, 1.05)	0.865
SAPS II	1.08 (1.02, 1.14)	**.013**	1.14 (1.03, 1.25)	**0.010**	1.12 (1.02, 1.22)	**0.014**	1.13 (1.05, 1.21)	**0.001**
Creatinine (per 0.1 unit log)	1.09 (0.85, 1.36)	.528	0.78 (0.50, 1.23)	0.286	0.72 (0.45, 1.15)	0.165	0.84 (0.60, 1.17)	0.303
(IGFBP7) * (TIMP-2) level (per 0.1 unit log)	1.36 (1.19, 1.54)	**<.001**	1.13 (0.99, 1.30)	0.077	1.07 (0.94, 1.22)	0.283	1.23 (1.10, 1.38)	**<0.001**
AUC (95%-CI)	0.881 (0.817, 0.944)		0.893 (0.817, 0.969)		0.825 (0.710, 0.940)		0.868 (0.793, 0.944)	
AUC (95%-CI) without (IGFBP7) * (TIMP-2) level	0.715 (0.617, 0.813)		0.843 (0.731, 0.956)		0.799 (0.661, 0.937)		0.805 (0.709, 0.902)	
p-value*	**<0.001**		0.067		0.279		**<0.001**	

*Likelihood-ratio test (comparing the fit of both models)

AKI, acute kidney injury; RRT, renal replacement therapy; IGFBP7, insulin-like growth factor-binding protein 7; TIMP-2, tissue inhibitor of metalloproteinase;

MAP, mean arterial pressure; AUC, area under the curve; CI, coincidence interval; OR, odds ratio.

Table 4 shows a multivariable logistic regression model with bedside postoperative parameters at the time of biomarker assessment alone and by adding the [TIMP-2]×[IGFBP7] test for predicting any AKI, AKI Stage 2 and 3, and the early use of RRT. By adding the [TIMP-2]×[IGFBP7] test to the postoperative clinical factors, the predictive power for AKI significantly improved (*P*<0.001); AUC 0.81 (95% CI 0.73, 0.90) increased to 0.89 (95% CI 0.83, 0.96). Values for AKI Stage 2 and 3 were AUC 0.87 (95% CI 0.78. 0.96) increasing to 0.89 (95% CI 0.81, 0.97), p = 0.002. The same effect was observed for the use of RRT, for which AUC 0.85 (0.69, 1.00) increased to AUC 0.86 (0.73, 0.99) (*P* = 0.035).

**Table 4:** Multivariable logistic regression model of bedside postoperative parameters at the time of biomarker assessment with and without the (TIMP-2)*(IGFBP7) for risk assessment of AKI, use of RRT, 28-days mortality and AKI 2,3.

	AKI		RRT		28 days mortality		AKI stage 2, 3	
Variable	OR (95%-CI)	p-value	OR (95%-CI)	p-value	OR (95%-CI)	p-value	OR (95%-CI)	p-value
Urine Output (per 1ml/Kg/h)	0.38 (0.15, 0.96)	**.041**	0.19 (0.03, 1.17)	.073	0.92 (0.33, 2.56)	0.872	0.12 (0.03, 0.56)	**0.007**
MAP (per 1mmHg)	1.02 (0.97, 1.06)	.459	1.02 (0.96, 1.09)	.478	1.01 (0.95, 1.07)	0.838	1.02 (0.97, 1.08)	0.407
Hemoglobin (per 1g/dl)	0.94 (0.71, 1.23)	.640	1.02 (0.70, 1.50)	.909	0.85 (0.58, 1.26)	0.419	0.84 (0.61, 1.15)	0.276
Fluid (per 1000ml)	1.00 (0.52, 1.95)	.994	1.02 (0.57, 1.81)	.961	1.25 (0.74, 2.10)	0.399	1.02 (0.53, 1.96)	0.953
Norepinephrine (per 0.1μg/kg/min)	3.06 (1.42, 6.60)	**.004**	1.36 (0.87, 2.13)	.176	1.34 (0.89, 2.04)	0.166	2.37 (1.07, 5.25)	**0.034**
(IGFBP7) * (TIMP-2) level (per 0.1 unit log)	1.33 (1.17, 1.52)	**<.001**	1.17 (1.01, 1.36)	.**043**	1.10 (0.96, 1.15)	0.167	1.20 (1.07, 1.36)	**0.003**
AUC (95%-CI)	0.892 (0.829, 0.956)		0.858 (0.729, 0.986)		0.810 (0.693, 0.927)		0.892 (0.812, 0.973)	
AUC (95%-CI) without (IGFBP7) * (TIMP-2) level	0.811 (0.725, 0.897)		0.847 (0.694, 1.000)		0.754 (0.571, 0.936)		0.868 (0.775, 0.962)	
p-value*	**<0.001**		**0.035**		0.167		**0.002**	

*Likelihood-ratio test (comparing the fit of both models)

AKI, acute kidney injury; RRT, renal replacement therapy; IGFBP7, insulin-like growth factor-binding protein 7; TIMP-2, tissue inhibitor of metalloproteinase; MAP, mean arterial pressure, AUC, area under the curve; CI, coincidence interval; OR, odds ratio.

## Discussion

Our study evaluated the use of the novel urinary cell-cycle arrest biomarkers [TIMP-2]×[IGFBP7] in patients with a high risk of AKI (at least one additional risk factor according to the KDIGO recommendation [19]) after major non-cardiac surgery.

The mean findings of our study were: 1) In addition to good prediction for moderate and severe AKI, as showed in the validation study, we found that any AKI (including and excluding stage 1) can be predicted in non-cardiac surgery patients with a predefined cutoff of 0.3. 2) Compared to the Sapphire study^16^, in which biomarkers were tested up to 12 hours after admission, risk stratification in our study occurred very early after surgery within the mean time of 245 minutes. These results illustrate the rapid responsiveness of the test. 3) In the multivariable model with perioperative risk factors and bedside clinical parameters, [TIMP-2]×[IGFBP7] test was the strongest predictor and significantly improved the detection of surgical patients with an increased risk of AKI. 4) Early cell cycle arrest after surgery may be associated with adverse outcome. 5) Patients after hepatic surgery showed increased median values of biomarkers, and only one patient developed AKI within 48 hours after surgery. These findings suggest that the early correction of hypovolemia, which is routinely performed postoperatively in this group of patients, may reverse cell cycle arrest and prevent the development of AKI.

In the present study, higher median values of [TIMP-2]×[IGFBP7] after admission were associated with an increased degree of renal injury within 48 hours after surgery. Patients with AKI stage 1 had the lowest median values, and patients requiring RRT had the highest median [TIMP-2]×[IGFBP7] test results. This association was crucial and showed that the degree of early cellular damage was associated with the severity of the functional defect occurring at a later stage.

Our results provide a new perspective on the performance of biomarkers in combination with perioperative parameters. Established risk factors for AKI are severity of the illness score, creatinine at admission, and age. In addition, we tested for the first time clinical parameters that help clinicians identify high-risk patients in the daily routine and that are associated with increased incidence or severity of AKI (urine output, hemoglobin level, mean arterial pressure, fluid balance, and use of vasopressors) [20–24]. In both models, use of the [TIMP-2]×[IGFBP7] test significantly improved AKI prediction. Moreover, the AUC for predicting AKI with urinary biomarker alone was higher than the prediction with perioperative risk factors in both clinical models (0.85 for biomarker alone vs. 0.72 for perioperative and 0.81 for bedside postoperative factors). Our results showed that the [TIMP-2]×[IGFBP7] test was the strongest predictor of AKI. Use of these biomarkers alone or in combination with established perioperative risk factors markedly improved early risk assessment.

The addition of the [TIMP-2]×[IGFBP7] test to clinical models for assessing the risk of 28-days mortality improved prediction in both models (AUC from 0.76 to 0.81 and from 0.80 to 0.83 respectively). However, this association was statistically not significant. The median value of the [TIMP-2]×[IGFBP7] test for patients dying within 28 days after surgery was lower than the median value for patients developing AKI stage 2–3 and for patients receiving RRT. This finding highlights the fact that the [TIMP-2]×[IGFBP7] test is primarily an AKI risk assessment tool. However, these findings support recent data suggesting that early cell cycle risk arrest in the kidneys may be associated with adverse outcome [25].

Interestingly, in the subgroup of patients after major hepatic surgery, relatively high median values of biomarkers of cellular injury contrasted with only one consecutive case of AKI within 48 hours after surgery. Application of a low CVP strategy is an effective and recommended method to reduce blood loss during liver surgery [26]. However, restrictive volume anesthesia is associated with an increased risk of acute renal failure in a postoperative setting [27]. Therefore, patients after liver resection are given higher amount of fluids in the early postoperative period to prevent prolonged perioperative hypoperfusion. In our study, the hepatic surgery subgroup had an unproportionally high mean positive fluid balance within first 24 hours postoperatively if compared to patients after cancer surgery with very similar baseline characteristics (1419 vs. 939 ml/24h). This fact may indirectly indicate that the early correction of hypovolemia may reverse early cell cycle arrest and prevent development of AKI.

Our study has several limitations. We conducted the study in one single surgical ICU. However, we believe that we examined a representative cohort of non-cardiac surgical patients. Our results showed higher rates of AKI (>42%) as well as early use of RRT (>10%). The high incidence of AKI − that may influence the predictive performance of biomarkers − was due to the study design that only included patients with a high risk of AKI. The previous two multicenter studies, however, have shown the ability of the [TIMP-2]×[IGFBP7] test to provide risk stratification for moderate and severe AKI in over 1100 critically ill patients with a prevalence of AKI, which was similar to that described in other literature reports. Moreover, another recent study has shown a very similar incidence of postoperative AKI of 39% in the cohort of 50 314 patients after major surgery [28]. Finally, we did not evaluate the long-term outcome (>28 days) in patients with cellular injury detected with [TIMP-2]×[IGFBP7] test. These issues need to be investigated in future studies.

In summary, we showed that the [TIMP-2]×[IGFBP7] test significantly improves the early prediction of any type of AKI in high-risk surgical patients. Due to its rapid responsiveness, the test extends the therapeutic window for renal protection measurements or future therapeutic interventions to prevent the development of AKI. Together with the known risk factors and bedside clinical parameters, this test helps clinicians more accurately identify patients who should consequently receive renal protective measures and escalation of care. Particularly postoperative hemodynamic optimization seems to decrease the risk of renal impairment in surgical patients [29]. This process could be started very early after surgery in patients with cellular stress diagnosed by means of urinary biomarkers. Future studies are required to determine whether early and more accurate risk stratification is consistently associated with better outcome for surgical patients with AKI.
